# Longitudinal impact of oral health on geriatric syndromes and clinical outcomes in community-dwelling older adults

**DOI:** 10.1186/s12877-021-02416-2

**Published:** 2021-09-04

**Authors:** Jihye Lim, Hyungchul Park, Heayon Lee, Eunju Lee, Danbi Lee, Hee-Won Jung, Il-Young Jang

**Affiliations:** 1 Department of Gastroenterology, Asan Medical Center, University of Ulsan College of Medicine, 88 Olympic-ro 43-gil, Songpa-gu, Seoul, Republic of Korea; 2 Department of Gastroenterology, Ilsan Paik Hospital, Inje University College of Medicine, Goyang, Republic of Korea; 3 Divison of Pulmonary, Critical Care, and Sleep Medicine, Department of Internal Medicine, Eunpyeong St. Mary’s Hospital, College of Medicine, The Catholic University of Korea, 1021 Tongil-ro, Jingwan-dong, Eunpyeong-gu, Seoul, South Korea; 4 Division of Geriatrics, Department of Internal Medicine, Asan Medical Center, University of Ulsan College of Medicine, 88 Olympic-ro 43-gil, Songpa-gu, Seoul, Republic of Korea; 5 PyeongChang Health Center and County Hospital, 11 Noseong-ro, Pyeongchang-eup, Pyeongchang-gun, Gangwon-do Gangneung, Republic of Korea

**Keywords:** Oral health, Frailty, Geriatrics

## Abstract

**Background:**

Oral health is essential for daily living and plays a pivotal role in overall health conditions and well-being. This study evaluated the impact of self-reported oral health on geriatric conditions, institutionalization, and mortality.

**Methods:**

This study analyzed the population of the Aging Study of Pyeongchang Rural Area that had undergone geriatric assessments between 2016 and 2017. The oral health status of the participants was determined using three items from the General Oral Health Assessment Index, and the participants were classified into three groups according to the total sum of the scores as good (3), fair (4–7), or poor (8–15). The outcomes were the incidence of geriatric syndromes at 2 years and the composite outcome of mortality and institutionalization.

**Results:**

Among the 1189 participants, 44.1 % were women, and the mean age of the study population was 75.0 years. Good, fair, and poor oral health were observed in 597 (50.2 %), 406 (34.1 %), and 186 (15.6 %) individuals, respectively. Worsening oral health status was associated with the incidences of various geriatric syndromes at follow-up, and these associations were attenuated after adjusting for baseline demographic and geriatric parameters. Similarly, the significant association between baseline oral health status and the incidence of the composite outcome was attenuated after adjusting for demographic and geriatric parameters.

**Conclusions:**

Oral health affected the geriatric health conditions in this prospective, longitudinal cohort of community-dwelling older adults. The correlations and interactions of oral health status with other functional parameters may deserve consideration as a geriatric domain.

**Supplementary Information:**

The online version contains supplementary material available at 10.1186/s12877-021-02416-2.

## Background

 Oral health is one of the most important domains of overall health among these charges in essential daily functions including nutritional intake and speaking. It also affects social interaction, mood, productivity, and self-esteem [1]. An estimated 3.5 billion people worldwide have dental problems, according to the 2017 Global Burden of Disease Study [2]. Oral health includes proper oral function without oral diseases such as edentulism, caries, xerostomia, and periodontal disease, assuring people to perform well on the aspects of physical, mental, and social functions without discomfort, pain, or embarrassment [3].

In older adults, factors such as cognitive impairment, decreased mobility, and limited socioeconomic resources may preclude receiving proper dental or medical care for oral health problems [1, 4, 5]. Furthermore, older adults with multiple comorbidities or polypharmacy are often restricted in receiving dental care due to concerns regarding procedure-related adverse outcomes [3]. Consequently, older individuals tend to use less dental services, resulting in the accumulation of untreated oral health problems that may consequently lead to a reduced quality of life and poorer nutritional status.

Previous cross-sectional and longitudinal studies showed that oral health status, as assessed according to the number of teeth, tongue pressure, or diverse questionnaires, is associated with frailty in older adults [6–9]. Frailty, defined as decreased physiological reserve and increased vulnerability to possible stressors, is associated with adverse health outcomes including falls, functional decline, higher healthcare use, and increased mortality in older adults [10, 11]. As oral health status can affect systemic inflammation and nutritional status that are also major mechanisms in the progression of frailty and functional decline, oral problems might be an important clinical issue among the geriatric domains in clinical care for older adults [12].

In the public health setting, studies suggested that overall health status including frailty can be improved by multicomponent intervention programs targeting physical performance, nutritional status, and geriatric functional domains [13–15]. However, less is known regarding the in-depth roles of oral health status on various geriatric domains and health outcomes over time, beyond the effects on nutritional status. Therefore, we used a three-item questionnaire to evaluate self-reported oral health to evaluate the associations between self-reported oral health and comprehensive geriatric syndromes to determine their longitudinal impacts on geriatric conditions and overall health outcomes including institutionalization and mortality in resource-limited public healthcare settings.

## Methods

### Study population

We analyzed records from the Aging Study of Pyeongchang Rural Area (ASPRA), a prospective population-based cohort focusing on frailty and geriatric syndromes of older, community-dwelling adults. The detailed design and baseline population characteristics of the ASPRA have been described previously [16]. The inclusion criteria for the study were (1) age ≥ 65 years; (2) enrolled in the National Healthcare Service and receiving public healthcare services; (3) self-ambulatory with or without assistive devices; (4) currently residing at home; and (5) able to provide informed consent either by themselves or by their family members. Individuals who were institutionalized or bed-ridden requiring nursing home-level care at home were excluded at the time of enrollment.

After the initial establishment of the study protocol in 2014, the study population was expanded to other areas in Pyeongchang county. Thus, this study included the records of 1,189 participants who had undergone baseline evaluations between 2016 and 2017. The cohort study was approved by the Institutional Review Board of Asan Medical Center, Seoul, Korea (Identification number: 2015 − 0673), and all participants provided written informed consent.

### Assessment of oral health status

We adopted a three-item questionnaire from the General Oral Health Assessment Index scale to assess oral health status [8]. The three questionnaire items were (1) “How often do you have problems in speaking clearly because of the status of your teeth or dentures” (assessment of physical function); (2) “How often do you limit the kinds or amounts of food you eat due to problems with your teeth or dentures” (assessment of pain or discomfort); and (3) “How often do you limit contact with people due to the condition of your teeth or dentures” (assessment of the psychosocial aspects of oral health) [8]. These items were scored with five grades ranging from 1 to 5 points, with higher scores indicating worse conditions. Based on a combined score ranging from 3 to 15 points, we established three oral health status groups: good (3 points), fair (4–7 points), and poor (8–15 points), considering the distributions of scores as shown below. We also assessed whether the participants were using removable dentures and assessed their sidedness (maxilla, mandible, or both) and functioning.

### Geriatric parameters

Trained nurses performed the comprehensive geriatric assessments (CGAs). Medical problems were assessed and multimorbidity was defined as having two or more of 11 diseases including diabetes, hypertension, asthma, chronic kidney disease, malignancy, chronic lung disease, myocardial infarction, angina, heart failure, stroke, and arthralgia [16]. We also counted the number of medications, with polypharmacy defined as the regular use of more than five medications. Activities of daily living (ADLs) for seven items including continence, bathing, dressing, toileting, transferring, eating, and washing of the hands and face, as well as instrumental activities of daily living (IADL) for 10 items including household chores, food preparation, going out a short distance, handling finances, grooming, laundry, shopping, transportation, managing own medications, and using the telephone, were assessed. The need for assistance for one or more items was considered a disability in ADL or IADL [17]. Cognitive status was assessed by the Korean version of the Mini-Mental State Examination, with cognitive dysfunction defined as scores of < 24 [18]. We used the Korean version of the Center for Epidemiological Studies depression scale to assess mood, with scores > 20 indicating the presence of depressive mood [19]. Nutritional status was assessed using the Mini-Nutritional Assessment-Short Form, with scores ≤ 11 indicating malnutrition [20]. Physical performance was assessed by the Short Physical Performance Battery (SPPB) to evaluate standing balance, walking speed, and five-time chair rise tests. The SPPB was scored from 0 to 12, with higher scores indicating better levels of function. We defined an SPPB total score of ≤ 9 as poor physical performance [21]. Muscle mass was measured by bioelectrical impedance analysis (InBody 620; InBody, Seoul, Republic of Korea), at 5, 50, and 500 kHz. Skeletal muscle index (SMI) was calculated as the appendicular skeletal muscle mass divided by the height^2^, with low muscle mass defined as SMI < 7.0 kg/m^2^ for men and < 5.7 kg/m^2^ for women [22]. Underweight was defined as a body mass index of ≤ 18.5 kg/m^2^.

Frailty status was assessed by the Cardiovascular Health Study frailty phenotype criteria [23, 24]. The frailty phenotype evaluated five domains: (1) exhaustion (poor endurance, a feeling that everything is an effort or moderate inability to get going for most of the time during the last week) [16]; (2) low activity level (lowest 20th percentile of physical activity according to the Korean version of the International Physical Activity Questionnaires Short Form [25]); (3) slowness (usual gait speed < 0.8 m/s in the 4-m walking test); (4) weakness (dominant hand grip strength < 26 kg for men and < 17 kg for women); and (5) weight loss (unintentional weight loss > 3 kg in the past 6 months) [26]. The number of positive items was scored and categorized as robust (0 points), prefrail (1–2 points), or frail (3–5 points).

### Outcome measures

The primary outcome measures were incidence of abnormality or impairment in development of disability in ADL or IADL, depressive mood, cognitive impairment, frailty, and low physical performance according to baseline oral health status in population of no baseline abnormality in specific domains, These outcomes were assessed annually with similar CGA components as in the baseline examination.

The secondary outcome was the composite of mortality and institutionalization to long-term care facilities due to functional decline, as reported by telephone interviews with participants and family members every 3 months until August 2020.

### Statistical analyses

We used one-way analysis of variance to assess differences in clinical parameters among the three oral health score groups. To assess the dose-response effects of oral health status on clinical parameters, we used linear regression analysis to calculate *P-*values for trends for categorical variables and Mann–Whitney or t-tests for continuous variables. To identify the associations between baseline oral health status and primary outcome at 2 years, we used logistic regression analysis to calculate the odds ratios. Analysis on incidence of the primary outcome measures were performed in population with no abnormality for each measure. To assess the associations between baseline oral health status and the secondary, composite outcome, we used Kaplan–Meier curves with log-rank tests and Cox regression analysis with hazard ratios. Two-sided *P*-values < 0.05 were considered statistically significant. The statistical analyses were performed using R (version 3.6.0, https://www.r-project.org).

## Results

### Characteristics of the study population

The mean age of the study population was 75.0 ± 6.2 years, and 44.1 % (524 of 1189) were women. The baseline characteristics of the participants are shown in Table 1. Good, fair, and poor oral health were reported by 597 (50.2 %), 406 (34.1 %), and 186 (15.6 %) of individuals. Individuals with poor oral health were more likely to be older; with low socioeconomic status; higher multimorbidity, polypharmacy, cognitive dysfunction, depression, dismobility, malnutrition, sarcopenia, and ADL/IADL disability; and have fall history in the past year (Table 1).

**Table 1:** Baseline characteristics according to self-reported oral health

	Good^a^	Fair^a^	Poor^a^	*P*-value	*P*-for trend
Variables	(*N* = 597)	(*N* = 406)	(*N* = 186)		
Gender (men)	255 (42.7 %)	200 (49.3 %)	69 (37.1 %)	0.014	0.699
Age (years)	73.6 ± 5.9	75.8 ± 6.0	78.0 ± 6.1	< 0.001	< 0.001
Living alone	144 (24.1 %)	130 (32.0 %)	63 (33.9 %)	0.005	0.002
Education level (years)	6.6 ± 4.0	5.8 ± 3.5	4.9 ± 3.1	< 0.001	< 0.001
Medical aid(monthly income < USD 500)	33 (5.5 %)	35 (8.6 %)	22 (11.8 %)	0.011	0.003
BMI (kg/m^2^)	25.0 ± 3.3	24.7 ± 3.7	24.8 ± 3.2	0.184	0.184
Multimorbidity	284 (47.6 %)	213 (52.5 %)	118 (63.4 %)	0.001	< 0.001
Hypertension	336 (56.3 %)	238 (58.6 %)	123 (66.1 %)	0.059	
Arthralgia	268 (44.9 %)	201 (49.5 %)	113 (60.8 %)	0.001	
Diabetes	111 (18.6 %)	73 (18.0 %)	55 (29.6 %)	0.002	
Heart failure	37 (6.2 %)	24 (5.9 %)	17 (9.1 %)	0.297	
Malignancy	28 (4.7 %)	25 (6.2 %)	13 (7.0 %)	0.394	
Polypharmacy	113 (18.9 %)	93 (22.9 %)	64 (34.4 %)	< 0.001	< 0.001
Cognitive dysfunction by MMSE	170 (28.5 %)	148 (36.5 %)	101 (54.3 %)	< 0.001	< 0.001
Depression by CES-D	298 (49.9 %)	248 (61.1 %)	146 (78.5 %)	< 0.001	< 0.001
SPPB score	8.7 ± 2.4	8.3 ± 2.4	7.2 ± 2.7	< 0.001	< 0.001
Malnutrition by MNA-SF	158 (26.5 %)	142 (35.0 %)	92 (49.5 %)	< 0.001	< 0.001
SMI (kg/m^2^)	16.1 ± 3.9	15.8 ± 4.3	14.6 ± 3.9	< 0.001	< 0.001
Frailty status by CHS				< 0.001	< 0.001
Robust	108 (18.1 %)	58 (14.3 %)	17 (9.1 %)		
Prefrail	401 (67.2 %)	259 (63.8 %)	109 (58.6 %)		
Frail	88 (14.7 %)	89 (21.9 %)	60 (32.3 %)		
ADL disability	75 (12.6 %)	86 (21.2 %)	52 (28.0 %)	< 0.001	< 0.001
IADL disability	119 (19.9 %)	121 (29.8 %)	77 (41.4 %)	< 0.001	< 0.001
Fall in the past year	102 (17.1 %)	93 (22.9 %)	56 (30.1 %)	< 0.001	< 0.001

Data presented as means ± standard deviations or numbers (%)

^a^According to the oral health item score, the groups were defined as good (3), fair (4–7), and poor (8–15)

*ADL* activities of daily living; *BMI* body mass index; *CES-D* Center for Epidemiologic Studies-Depression; *CHS* Cardiovascular Health Study; *IADL* Instrumental Activities of Daily Living; *MMSE* Mini-Mental State Examination; *MNA-SF* Mini Nutritional Assessment-Short Form; *SMI* Skeletal Muscle Index; *SPPB* Short Physical Performance Battery

Physical difficulties when speaking were reported by 295 (24.9 %, item 1) individuals, while 544 felt discomfort when eating in (45.8 %, item 2) and 58 reported psychosocial distress when in contact with others (4.9 %, item 3), as shown in Additional file 1, Supplementary Table 1. The mean and standard deviation of the thee item-based total score was 5.1 ± 2.7.

A total of 52.2 % (621 of 1189) of participants used removable dental prosthesis, among whom 81.3 % (505/621) used both-sided dentures. Among participants not currently using prostheses, 15.7 % (89/568) needed dentures, while 8.2 % (51/621) of prosthesis users felt that dentures were unnecessary. The participants using both-sided dentures showed the highest overall scores for the questionnaire, followed by one-sided denture users and non-denture users in Supplementary Table 2 (5.7 ± 2.9 vs. 5.1 ± 2.6 vs. 4.6 ± 2.5, *P* < 0.001).

### Impacts of self-reported oral health on common geriatric conditions at 2 year

Poor oral health was associated with the deterioration of geriatric conditions and frailty. We evaluated the new occurrence of each variable during the 2 years of follow-up among the participants who did not have each geriatric syndrome at baseline. As shown in Table 2, baseline poor oral health was associated with increased risks of developing cognitive dysfunction, depression, low physical performance, disability (ADL or IADL), and frailty (Table 2). However, after controlling for demographic and geriatric parameters no significant association could be shown.

**Table 2:** Oral health status and incidence of geriatric syndromes at 2 years (logistic regression analysis)

	Model 1	Model 2	Model 3
	OR (95 % CI)	OR (95 % CI)	OR (95 % CI)
Underweight			
Good	Reference	Reference	Reference
Fair	0.74 (0.18–2.96)	0.57 (0.14–2.33)	0.51 (0.13–2.00)
Poor	2.18 (0.61–7.8)	1.47 (0.38–5.67)	1.54 (0.44–5.43)
Low muscle mass			
Good	Reference	Reference	Reference
Fair	1.41 (0.72–2.76)	1.52 (0.74–3.14)	0.91 (0.56–1.48)
Poor	1.78 (0.79–4.03)	1.02 (0.41–2.53)	1.26 (0.71–2.22)
Cognitive dysfunction			
Good	Reference	Reference	Reference
Fair	1.06 (0.68–1.64)	0.99 (0.62–1.58)	1.00 (0.73–1.39)
Poor	2.10 (1.23–3.60)	1.36 (0.76–2.45)	1.45 (0.98–2.14)
Depression			
Good	Reference	Reference	Reference
Fair	2.06 (1.19–3.57)	2.11 (1.20–3.71)	1.40 (0.89–2.22)
Poor	3.11 (1.66–5.85)	2.68 (1.39–5.19)	1.77 (1.04–2.99)
Low physical performance			
Good	Reference	Reference	Reference
Fair	1.42 (1.01–2.01)	1.23 (0.85–1.79)	1.08 (0.85–1.39)
Poor	2.23 (1.31–3.79)	1.55 (0.88–2.72)	1.20 (0.86–1.68)
Disability			
Good	Reference	Reference	Reference
Fair	1.17 (0.81–1.69)	1.15 (0.78–1.69)	0.96 (0.74–1.25)
Poor	1.79 (1.06–3.03)	1.50 (0.86–2.61)	1.16 (0.83–1.63)
Multimorbidity			
Good	Reference	Reference	Reference
Fair	0.88 (0.51–1.54)	0.91 (0.51–1.62)	0.80 (0.56–1.13)
Poor	1.00 (0.46–2.17)	0.84 (0.37–1.88)	0.83 (0.51–1.37)
Frailty			
Good	Reference	Reference	Reference
Fair	1.49 (1.19–1.87)	1.24 (0.97–1.59)	1.27 (0.92–1.76)
Poor	3.20 (2.46–4.17)	2.08 (1.55–2.78)	1.40 (0.95–2.07)

Model 1: unadjusted analysis

Model 2: adjusted for age and gender

Model 3: adjusted for age, gender, baseline medical aid, multimorbidity, polypharmacy, cognitive dysfunction, depression, low physical performance, ADL or IADL disability, and low muscle mass

*CI* confidence interval; *OR* odds ratio

### The composite outcome of mortality and institutionalization and oral health

**Fig. 1:**
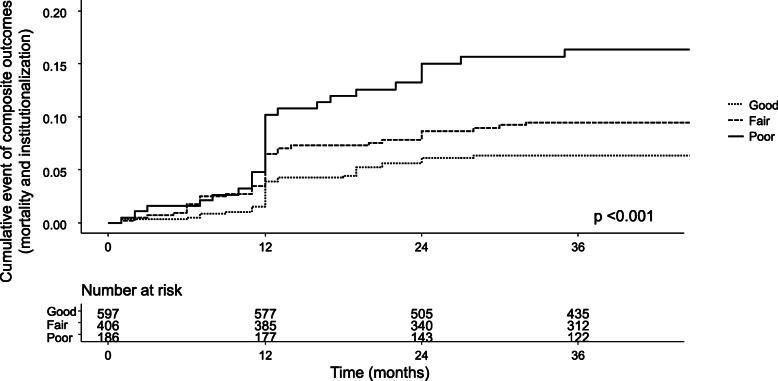
Kaplan–Meyer analysis of the composite outcome (institutionalization and death) by self-reported oral health groups

During the median follow-up of 40 months (range, 35–47), 50 individuals were institutionalized and 52 died. The overall probability of the composite outcome comprising institutionalization or mortality according to self-reported oral perception is shown in Fig. 1. People with good oral health had a better composite outcome compared to that for individuals with fair or poor oral health (good vs. fair vs. poor; 96.6 % vs. 94.8 % vs. 95.2 % at 1 year and 72.9 % vs. 76.8 % vs. 65.6 % at 3 years; log-rank *P* < 0.001). In an unadjusted Cox regression analysis, worsening self-reported oral perception was significantly associated with the incidence of the composite outcome (Table 3). After adjusting for age, gender, and baseline geriatric parameters, the correlation between baseline oral health and the composite outcome was attenuated.

**Table 3:** Oral health status and institutionalization or death by Cox regression analysis

	Model 1	Model 2	Model 3
	OR (95 % CI)	OR (95 % CI)	OR (95 % CI)
Good	Reference	Reference	Reference
Fair	1.53 (0.97–2.42)	1.12 (0.71–1.78)	0.99 (0.62–1.58)
Poor	2.68 (1.64–4.37)	1.52 (0.92–2.53)	1.19 (0.71–2.00)

Model 1: unadjusted analysis

Model 2: adjusted for age and gender

Model 3: adjusted for baseline medical aid, multimorbidity, polypharmacy, cognitive dysfunction, depression, low physical performance, ADL or IADL disability, and low muscle mass

*CI* confidence interval; *OR* odds ratio

## Discussion

In this prospective cohort study of older participants representing the general rural population of Korea, a worsening self-reported state of oral health was associated with deteriorating geriatric clinical parameters at 2 years and adversely affected the composite outcome of death and institutionalization. However, these associations were attenuated after adjusting for demographic and geriatric parameters. While most previous studies assessed oral health status as a predictor of future undernutrition in older adults, this study comprehensively evaluated the interaction between oral health status at baseline and for future geriatric functional parameters.

In our study, individuals with poor self-reported oral health were more likely to be malnourished and sarcopenic, consistent with literature supporting the importance of oral health parameters such as the number of teeth, salivary secretion, and masticatory ability in maintaining essential daily nutrition [27]. Individuals with poor oral health have difficulty in chewing vegetables, fruit, and meat [7] and thus, may consume comparatively easy-to-swallow but less nutritious food such as mashed or blended foods with high sugar or fat content [1, 9, 28, 29]. Moreover, in our study, participants with poor oral health had a higher rate of baseline multimorbidity, consistent with previous studies supporting the association of poor oral condition with systemic diseases including diabetes, cardiovascular disease, pulmonary infection, kidney disease, and even dementia [30–34]. Possible explanations for these associations include systemic inflammatory responses due to periodontitis or oral abscess causing comorbidity, and the fact that oral disease and comorbidities share risk factors such as alcohol, smoking, and unhealthy diet [35]. Unsatisfactory oral health also can adversely affect mood, as observed in the present study. Individuals tend to be socially withdrawn when feeling embarrassed to eat or communicate with others due to inaccurate pronunciation or aesthetic appearance from a lack of teeth [36]. These factors may negatively affect mental health, lower self-esteem, and cause depression [36, 37].

In addition to these cross-sectional associations of oral health and geriatric parameters, we also observed the longitudinal impacts of baseline oral health on the future incidence of geriatric syndromes and composite outcome, although the impacts of oral health status on the incidences of the clinical outcomes were attenuated by including baseline geriatric parameters. Frailty, a clinically recognizable state of vulnerability with decreased physiological reserve occurring with human aging, is associated with falls, disabilities, treatment-related adverse outcomes, and even mortality [38]. The risk factors for frailty and frailty progression include socio-demographic, physical, biological, psychological, and lifestyle factors [39, 40]. Previous studies revealed the impact of oral health on physical (weight and muscle loss, disability, and mobility), biological (inflammation), psychological (depression and cognition), and lifestyle (food intake) factors [41–44]. Our results also showed the relationship between oral and general health. With previously reported evidence suggesting frailty as an aging phenomenon correlated with global functional parameters [16, 45], these various conditions may lead to longitudinal deterioration of frailty, resulting multifaceted deterioration across geriatric parameters.

We used three self-reported items to evaluate oral health. In resource-limited, real-world clinical practices, the use of comprehensive oral health assessments might be less feasible. We selected these three items to reflect the various facets of oral health including physical function in speaking (item 1), pain and discomfort (item 2), and psychosocial/psychological aspects (item 3) [8]. These limited items captured the cross-sectional correlations and longitudinal clinical relevance of baseline oral health status. It would be beneficial to do brief oral assessments in caring for geriatric populations.

Recent reports indicate that frailty and geriatric syndromes can be managed even in resource-scarce rural communities through the applicate of multicomponent intervention programs including exercise; nutrition; and geriatric management of potentially inappropriate medications, chronic diseases, cognitive- and mood problems [46, 47]. The combination of the observations from the present study with those of previous reports allows the future assessment of the beneficial effects of community-based programs targeting older populations to improve oral health status on frailty and geriatric outcomes on top of dental health status *per se*.

Our study has some limitations. First, our study population was limited to a rural country of South Korea. Therefore, our results may not be generalized to other regions and ethnicities, although previous reports from the same population showed comparable characteristics to those in Korean rural-dwelling older populations [16]. Second, medically objective oral health evaluations were not performed. Further studies are warranted that include physician-assessed oral health, with some interventional attempts to assess the possible protective effects of dental care in older populations to improve geriatric outcomes. Third, while the statistical associations between oral health status and incidence of geriatric syndromes or the composite outcome were attenuated in multivariate analysis, positive trends for worsening baseline oral health toward increased likelihoods of the negative outcomes were observed. Because the independent impact of oral health on geriatric outcomes could not be confirmed in our cohort, future studies in larger populations might be informative.

## Conclusions

In conclusion, the results of our study showed the clinical impacts of oral health on geriatric health conditions in a prospective, longitudinal cohort of community-dwelling older adults. Rather than a mere predictor of nutritional status, oral health status can also be a geriatric parameter associated with baseline and the future burden of geriatric syndromes. More vigilance regarding the oral health status for adequate therapeutic approaches may lead to better clinical outcomes in caring for older adults at risk of frailty. In that way, it would help older adults to have healthy longer life.

## Supplementary Information

Additional file 1: **Supplementary Table S1**. The prevalence and degree of difficulty for each item**Supplementary Table S2**. Baseline characteristics according to dental prosthesis

## Data Availability

The raw data of the current study are not publicly available due to the protection of the participants personal information but are available from the corresponding author on reasonable request.

## Abbreviations

ADL: Activities of daily living
ASPRA: Aging Study of Pyeongchang Rural Area
IADL: Instrumental activities of daily living
CGA: Comprehensive geriatric assessment
OR: Odds ratio
SPPB: Short Physical Performance Battery
SMI: Skeletal muscle index
