# Supplementary material for: Longitudinal impact of oral health on geriatric syndromes and clinical outcomes in community-dwelling older adults
Source: BMC Geriatr. 2021 Sep 4;21:482. doi: 10.1186/s12877-021-02416-2 (PMC8418721; doi:10.1186/s12877-021-02416-2)
Supplement: Supplementary file 1 — Additional file 1: Supplementary Table S1. The prevalence and degree of difficulty for each itemSupplementary Table S2. Baseline characteristics according to dental prosthesis [file 12877_2021_2416_MOESM1_ESM.doc]

**Longitudinal impact of oral health on geriatric syndromes and clinical outcomes**

**in community-dwelling older adults**

Jihye Lim, Hyungchul Park, Heayon Lee, Eunju Lee, Danbi Lee,

Hee-Won Jung, Il-Young Jang

**Table contents**

**Supplementary Table 1**

**Supplementary Table 2**

**Supplementary Table 1.** The prevalence and degree of difficulty for each item

|  | **Item 1*** | **Item 2*** | **Item 3*** |
| --- | --- | --- | --- |
| Never | 893 (75.1%) | 645 (54.2%) | 1131 (95.1%) |
| Sometimes | 55 (4.6%) | 82 (6.9%) | 16 (1.3%) |
| Often | 80 (6.7%) | 149 (12.5%) | 18 (1.5%) |
| Frequently | 49 (4.1%) | 60 (5.0%) | 9 (0.8%) |
| Always | 112 (9.4%) | 253 (21.3%) | 15 (1.3%) |

Item 1: How often do you have problems with speaking clearly?

Item 2: How often do you limit the kinds or amounts of food you eat?

Item 3; How often do you limit your contact with people?

**Supplementary Table 2.** Baseline characteristics according to dental prosthesis

|  | **Without denture** | **One-sided denture** | **Both-sided denture** | ***P*-value** |
| --- | --- | --- | --- | --- |
| Variables | (N=568) | (N=116) | (N=505) |  |
| Gender (men) | 283 (49.8%) | 61 (52.6%) | 180 (35.6%) | <0.001 |
| Age (years) | 73.1 ± 5.8 | 74.1 ± 5.8 | 77.3 ± 6.0 | <0.001 |
| Living alone | 130 (22.9%) | 26 (22.4%) | 181 (35.8%) | <0.001 |
| Education level (years) | 7.1 ± 4.1 | 5.8 ± 3.4 | 4.9 ± 3.0 | <0.001 |
| Medical aid  (monthly income <USD 500) | 31 (5.5%) | 6 (5.2%) | 53 (10.5%) | 0.005 |
| BMI (kg/m^2^) | 25.0 ± 3.5 | 24.9 ± 3.3 | 24.8 ± 3.4 | 0.232 |
| Multimorbidity | 256 (45.1%) | 56 (48.3%) | 303 (60.0%) | <0.001 |
| Hypertension | 310 (54.6%) | 66 (56.9%) | 321 (63.6%) | 0.011 |
| Arthralgia | 229 (40.3%) | 50 (43.1%) | 303 (60.0%) | <0.001 |
| Diabetes | 110 (19.4%) | 27 (23.3%) | 102 (20.2%) | 0.631 |
| Heart failure | 31 (5.5%) | 8 (6.9%) | 39 (7.7%) | 0.323 |
| Malignancy | 27 (4.8%) | 9 (7.8%) | 30 (5.9%) | 0.384 |
| Polypharmacy | 117 (20.6%) | 23 (19.8%) | 130 (25.7%) | 0.098 |
| Cognitive dysfunction by MMSE | 25.4 ± 4.3 | 25.0 ± 3.5 | 23.2 ± 4.6 | <0.001 |
| Depression by CES-D | 282 (49.6%) | 70 (60.3%) | 340 (67.3%) | <0.001 |
| SPPB score | 8.8 ± 2.4 | 8.5 ± 2.4 | 7.8 ± 2.5 | <0.001 |
| Malnutrition by MNA-SF | 175 (30.8%) | 32 (27.6%) | 185 (36.6%) | 0.055 |
| SMI (kg/m^2^) | 16.5 ± 4.1 | 16.4 ± 3.9 | 14.7 ± 3.8 | <0.001 |
| Frailty status by CHS |  |  |  | <0.001 |
| Robust | 115 (20.2%) | 18 (15.5%) | 50 (9.9%) |  |
| Prefrail | 381 (67.1%) | 71 (61.2%) | 317 (62.8%) |  |
| Frail | 72 (12.7%) | 27 (23.3%) | 138 (27.3%) |  |
| Sum of oral health item score | 4.6 ± 2.5 | 5.1 ± 2.6 | 5.7 ± 2.9 | <0.001 |
| ADL disability | 76 (13.4%) | 18 (15.5%) | 119 (23.6%) | <0.001 |
| IADL disability | 114 (20.1%) | 28 (24.1%) | 175 (34.7%) | <0.001 |
| Fall in the past year | 113 (19.9%) | 23 (19.8%) | 115 (22.8%) | <0.001 |

Data presented as means ± standard deviations or numbers (%)

ADL, activities of daily living; BMI, body mass index; CES-D, Center for Epidemiologic Studies-Depression; CHS, Cardiovascular Health Study; IADL, Instrumental Activities of Daily Living; MMSE, Mini-Mental State Examination; MNA-SF, Mini Nutritional Assessment-Short Form; SMI, Skeletal Muscle Index; SPPB, Short Physical Performance Battery
